# Blepharoptosis Associated With the Wearing of Hard Contact Lenses in Keratoconic Eyes

**DOI:** 10.7759/cureus.74529

**Published:** 2024-11-26

**Authors:** Kensei Hoshi, Kazuhiko Dannoue, Hiroyuki Namba, Junko Yoshida, Tomohiko Usui

**Affiliations:** 1 Ophthalmology, International University of Health and Welfare, Chiba, JPN; 2 Ophthalmology, Dannoue Eye Clinic, Kawasaki, JPN; 3 Ophthalmology, International University of Health and Welfare, Tokyo, JPN

**Keywords:** blepharoptosis, hard contact lenses, keratoconus, levator function, margin reflex distance

## Abstract

Keratoconus is a condition that causes progressive thinning and anterior protrusion of the cornea. Because of its irregular astigmatism, mild to moderate keratoconus is corrected with hard contact lenses (HCLs), but blepharoptosis due to the long-term wearing of HCLs is often a problem. In this study, we investigated blepharoptosis in HCL wearers with keratoconus. Thirty-seven keratoconic eyes were enrolled and divided into several groups according to their wearing history or age. Eyelid margin distance (EMD), margin light reflex distance-1 (MRD-1), and levator function (LF) were measured using a distantometer or anterior segment optical coherence tomography (AS-OCT). The EMD decreased with years of wear but showed no correlation with age; the MRD-1 decreased with years of wear and with older age; the LF showed no relationship with either years of wear or age. In the classification of ptosis, there was no severe ptosis in the present sample. The long-term wearing of HCLs is likely to cause blepharoptosis even in keratoconic eyes.

## Introduction

Keratoconus is a progressive disorder that causes irregular astigmatism due to thinning and protrusion of the corneal stroma [1-3]. According to a meta-analysis of more than seven million patients in 15 countries, the prevalence of the disease is estimated as 1.38 per 1000 people (approximately one in 700-800 people) worldwide [4]. Although eye rubbing is a risk factor for the development of keratoconus and has been linked to atopic dermatitis, Down syndrome, and sleep apnea syndrome, the mechanism of its development is not yet known [1]. The treatment for keratoconus is corneal transplantation in advanced severe cases, and corneal crosslinking is selected for mild or moderate cases in progress [1].

Hard contact lenses (HCLs) are one of the options for refractive correction [5]. Although HCLs are very beneficial in that they allow refractive correction even in the presence of a certain degree of irregular astigmatism or higher-order aberration, there are also disadvantages associated with them, such as chronic inflammation of the ocular surface, epithelial damage to the corneal and conjunctival epithelium, and the risk of infection due to improper use. Blepharoptosis has also been reported as one of the complications associated with the long-term wearing of HCLs [6-9]. Prolonged soft contact lens wearers also develop blepharoptosis, but HCL wearers are 17 times more likely to develop blepharoptosis than non-contact lens wearers [10]. Blepharoptosis not only causes esthetic problems but also the impairment of visual functions. Blepharoptosis impairs upward vision and has been reported to cause visual field defects as measured with the Goldmann manual kinetic perimetry [11] or static automated perimetry [12]. It has also been noted that the limitation of light inflow into the pupil due to ptosis may cause impairment of contrast sensitivity [13]. Furthermore, since the upper eyelid is directly attached to the cornea, blepharoptosis can affect the shape of the cornea [14]. We previously reported on the effect of blepharoptosis on corneal astigmatism [15]. Although this complication is well-known in patients with myopia who wear HCLs, to the best of our knowledge, the relationship between keratoconus who wear HCLs and HCL-induced blepharoptosis is not yet understood. Therefore, in this study, we tried to evaluate blepharoptosis in keratoconus patients who ordinarily wear HCLs.

## Materials and methods

The enrolled subjects were 38 eyes of 38 patients who were diagnosed as having keratoconus and wearing HCLs who visited the International University of Health and Welfare Narita Hospital, Japan. The mean age was 41.2 ± 14.7 years. The average duration of wearing HCLs was 19.5 ± 13.1 years. Of the 38 eyes, 28 eyes had worn an HCL every day, and 10 eyes had worn an HCL more than four days a week. The eyes with corneal crosslinking, intrastromal corneal ring implantation, or intraocular lens implantation were not included. Patients were excluded from this study if they had a corneal pathology other than keratoconus (e.g., pterygium). Subjects with missing or insufficient data were also excluded.

In this study, we measured the eyelid margin distance (EMD), margin reflex distance-1 (MRD-1), and levator function (LF). The EMD was measured from the middle upper eyelid to the middle lower eyelid with a ruler (Distantometer, Handaya, Tokyo, Japan). The MRD-1 was measured from the pupil center to the middle upper eyelid with a distantometer [16]. The MRD-1 was also measured using anterior segment optical coherence tomography (AS-OCT; SS-2000, CASIA2, Tomey, Nagoya, Japan). In AS-OCT, the capture mode was sagittal (B-scan range was 16 mm, C-scan range was 16 mm, the number of A/B scans was 800, and the number of B/C scans was 32, respectively). For the analysis, the measurement position was moved to the center of the pupil, and the distance from the pupil center to the middle upper eyelid was manually analyzed using the built-in calipers with the angle set at 90 degrees [17]. The EMD and MRD-1 were measured with the eyelids naturally open. The LF was measured with a distantometer as the distance that the upper eyelid moved from its position when the subject was looking downward to its position when the subject was looking upward. The measurement of LF was performed by gently holding the upper part of the eyebrow with a finger to prevent elevation using the frontalis muscle.

We compared these parameters with the HCL-wearing periods or age. Regarding the HCL wearing periods, the patients were divided into three groups: 10 years or less (n=10), 11 to 20 years (n=11), and 21 years or more (n=17). Regarding age, the patients were also divided into three groups: 10-20s (n=12), 30-40s (n=15), and 50s and older (n=11). Statistical analyses were performed using the Kruskal-Wallis test. After the Kruskal-Wallis test confirmed the significant difference, the Steel-Dwass test with Bonferroni correction was used to compare the two groups. The data were analyzed using EZR statistical analysis software (Saitama Medical Center, Jichi Medical University, Saitama, Japan, https://www.jichi.ac.jp/saitama-sct/SaitamaHP.files/statmedEN.html) [18], which is a graphical user interface for R (The R Foundation for Statistical Computing, Vienna, Austria). More precisely, it is a modified version of R Commander (version 1.6-3) that was designed to add statistical functions frequently used in biostatistics. All P-values of 0.05 or less were considered statistically significant. 

## Results

The results are summarized in Table 1. The average EMD was 8.67 ± 1.03 mm for those who had worn HCLs for 10 years or less, 8.00 ± 1.05 mm for those who had worn HCLs for 11 to 20 years, and 6.50 ± 1.71 mm for those who had worn HCLs for 21 years or more. There were statistically significant differences between those who had worn HCLs for 10 years or less and those who had worn HCLs for 21 years or more (P<0.01). The average EMD was also 8.00 ± 0.87 for those who were 29 years old or younger, 8.10 ± 1.05 for those who were 30 to 49 years old, and 6.90 ± 1.75 for those who were 50 years old or older. There were no significant differences among these age groups.

**Table 1:** The EMD, MRD-1, LF in patients with keratoconus who wear HCLs HCL: hard contact lens; EMD: eyelid margin distance; MRD-1: margin reflex distance-1; LF: levator function; AS-OCT: anterior segment optical coherence tomography *:P<0.05

		EMD	MRD by the distantometer	MRD by the AS-OCT	LF
HCL wearing periods (years)	10 or less (n=10)	8.80±1.06 *	3.75±0.54 *	3.56±1.12	15.10±3.75
	11 to 20 (n=11)	8.18±0.84	3.59±0.63	3.43±0.64	14.95±1.80
	21 or more (n=17)	7.32±2.14 *	3.08±1.08 *	3.06±0.95	13.53±3.21
Age (years)	10s – 20s (n=12)	8.17±0.75	3.75±0.66 *	3.62±1.04	15.08±1.73
	30s – 40s (n=15)	8.06±1.63	3.47±0.67	3.27±0.74	14.77±3.34
	50s – 70s (n=11)	6.95±1.27	2.91±0.63 *	2.94±0.48	13.55±1.81
					unit; mm

The average MRD-1 using a distantometer was 3.72 ± 0.57 mm for those who had worn HCLs for 10 years or less, 3.50 ± 0.63 mm for those who had worn HCLs for 11 to 20 years, and 2.36 ± 0.38 mm for those who had worn HCLs for 21 years or more. There were statistically significant differences in MRD-1 between those who had worn HCLs for 10 years and those who had worn HCLs for 21 years or more (P < 0.01) and between those who had worn HCLs for 11 to 20 years and those who had worn HCLs for 21 years or more (P < 0.05). The average MRD-1 using a distantometer was also 3.79 ± 0.70 for those who were 29 years old or younger, 3.20 ± 0.67 for those who were 30 to 49 years old, and 2.50 ± 0.61 for those who were 50 years old or older. There were no significant differences among these age groups.

The average MRD-1 using AS-OCT was 3.49 ± 1.13 mm for those who had worn HCLs for 10 years or less, 3.47 ± 0.73 mm for those who had worn HCLs for 11 to 20 years, and 2.83 ± 0.22 mm for those who had worn HCLs for 21 years or more. There were no significant differences among these groups based on wearing periods. The average MRD-1 using AS-OCT was also 3.79 ± 1.05 for those who were 29 years old or younger, 3.06 ± 0.74 for those who were 30 to 49 years old, and 3.00 ± 0.48 for those who were 50 years old or older. There were no significant differences among these age groups.

The average LF was 14.00 ± 1.50 mm for those who had worn HCLs for 10 years or less, 13.75 ± 1.54 mm for those who had worn HCLs for 11 to 20 years, and 12.86 ± 2.34 mm for those who had worn HCLs for 21 years or more. There were no significant differences among these groups based on wearing periods. The average LF was also 14.14 ± 1.68 for those who were 29 years old or younger, 13.45 ± 1.80 for those who were 30 to 49 years old, and 13.00 ± 2.12 for those who were 50 years or older. There were no significant differences among these age groups as well.

## Discussion

Blepharoptosis is a well-known complication of long-term wear of HCLs [5-8], but its etiology is not yet well understood. Multiple factors have been proposed, including fibrosis of the Müller muscle due to repeated friction [8], inflammation of the upper eyelid tissue [19], and/or thinning of the elevator tendon membrane and detachment from the eyelid plate due to the outward pulling motion of the upper eyelid during insertion or removal [20]. It is conceivable that the same insertion and removal methods are used in keratoconus as in normal myopia. It is quite possible that inflammation and friction caused by HCLs may also occur in patients with keratoconus.

Regarding HCL-induced blepharoptosis for normal myopic eyes, Bosch et al. reported that MRD-1 was 0.5 mm lower in HCL wearers who had worn them for more than 10 years and with a mean wearing duration of 16.3 years compared to non-wearing patients [6]. In this study, the average MRD-1 values obtained using a distantometer and AS-OCT were 2.88 mm and 3.02 mm, respectively, in patients with keratoconus who had worn HCLs for more than 10 years. Furthermore, as shown in the results of the present study, the longer the patients wore HCLs and the older they became, the smaller the MRD-1 value was (Table 1). Our data indicate that even keratoconic eyes could develop blepharoptosis with the long-term wearing of HCLs.

In the report by Watanabe et al., the degree of blepharoptosis was classified into four groups according to the MRD-1 value: grade 1, no ptosis (2.8 mm or more); grade 2, mild (1.5 mm to 2.8 mm); grade 3, moderate (0 mm to 1.5 mm); and grade 4, severe (0 mm or less) [8]. Applying this classification to the present study, six eyes had grade 2, 0 had grade 3, 0 had grade 4, and 32 had grade 1 ptosis as measured by the ruler, while 12 eyes had grade 2, 0 had grade 3, 0 had grade 4, and 26 had grade 1 ptosis as measured by the AS-OCT. Thus, there were no severe cases, and most of the cases were mild. The average grade was mild for both the ruler and the AS-OCT measurements for the subjects who were over 50 years old. Otherwise, according to a paper from Watanabe et al., the average grade was already above the moderate level of grade 3 for those who were 40 years old, and the average grade was 3.3 for those in their 50s and 3.5 for those in their 60s [9]. These authors also showed that 10 years of wearing HCLs provided a strong risk for the progression of blepharoptosis [8]. However, our data indicating that there were no significant differences in any of the parameters between the two groups of patients who had worn HCLs for 10 years or less and those who had worn HCLs for 11 to 20 years or less suggest that HCL-induced ptosis may be less severe in patients with keratoconus. Thus, the degree of blepharoptosis from HCL use in keratoconic eyes may be less severe than the ptosis caused by HCL use in normal myopic eyes.

Several differences between normal corneas and keratoconus are relevant to the use of HCL. The fitting of the HCL on the ocular surface is different for normal and keratoconus corneas. In normal corneas, the posterior surface of the HCL and the corneal curvature match not only at the center of the lens but also at the periphery, but such fitting is difficult in keratoconic eyes due to the non-uniformity of the corneal curvature. Therefore, spherical HCLs with a larger size than usual are often used for the HCL prescription for keratoconus [21]. These differences in fitting methods and HCL size may have different effects on the eyelid. The fitting of the HCLs in keratoconic eyes is such that it is supported by the lower eyelid and is expected to have little impact on the upper eyelid, such as friction. Therefore, there is little effect on the Müller muscle, which can be seen from the fact that the LF does not decrease (Table 1). 

The structure of the eyelid may also differ between keratoconus and normal myopic eyes. For example, keratoconus is associated with floppy eyelid, a condition in which the upper eyelid is not tight and rubbery and is easily rolled out [22]. Floppy eyelid is more common in obese men in their 40s and 50s and is often associated with obstructive sleep apnea [22]. Pathologically, a marked decrease in the elastin content of the eyelid plate has been noted [22]. Thus, other eyelid changes specific to keratoconus may also occur, although we did not investigate the presence or absence of floppy eyelids in this study. 

In this study, MRD-1 was measured using both a distantometer and AS-OCT. While the method of using a ruler to measure the degree of blepharoptosis has the advantage of being simple and not requiring expensive equipment, its disadvantages are that it is difficult to make detailed measurements because the measurement is in increments of 1 mm, and the variation among examiners may be large. On the other hand, AS-OCT enables accurate and objective measurements with the smallest unit of measurement being 0.01 mm because of its high resolution. Ogasawara et al. previously reported a simple and highly reproducible measurement of MRD-1 using AS-OCT [17].

As a limitation of this study, the study had a relatively small sample size. We were not able to confirm the detailed daily duration of HCL use and care or the size of the HCLs. Another limitation is that this is a retrospective study with small samples. The timing of the onset of blepharoptosis and MRD-1 on the first occasion of contact lens use was completely unknown. A further limitation is that the enrolled subjects in this study were all Japanese. The normal values of EMD, MRD-1, and LF are expected to differ between the Caucasian and Asian populations. There may also be differences in the anatomy of the eyelid. In fact, racial differences in eyelid fatty degeneration have been reported [23-25].

## Conclusions

This is the first report of HCL-induced blepharoptosis specifically in patients with keratoconus. The longer the wearing period, the lower the MRD-1 value, and the more likely the patient was to develop blepharoptosis in their keratoconic eyes. A literature review suggests that ptosis due to HCL in keratoconus eyes may be mild, but further studies are needed to reach a conclusion.
